# Management of pediatric forearm fractures: what is the best therapeutic choice? A narrative review of the literature

**DOI:** 10.1007/s12306-020-00684-6

**Published:** 2020-10-14

**Authors:** G. Caruso, E. Caldari, F. D. Sturla, A. Caldaria, D. L. Re, P. Pagetti, F. Palummieri, L. Massari

**Affiliations:** Orthopedic and Traumatology Unit, Sant’Anna University Hospital, via Aldo Moro no. 8, 44124 Cona, FE Italy

**Keywords:** Forearm, Fractures, Pediatric, Trauma, Casts, Surgery, Conservative treatment

## Abstract

**Purpose:**

This narrative review intends to summarize the most important and relevant data on diagnosis and treatment of pediatric forearm fractures and to describe the characteristics and advantage of each therapeutic option.

**Methods:**

We conducted a literature research considering peer-reviewed papers (mainly clinical trials or scientific reviews) using the string “forearm fracture AND epidemiology” or “forearm fracture AND diagnosis or “ forearm fracture AND treatment” or “forearm fracture AND casting” or “forearm fracture AND surgery”. Studies were identified by searching electronic databases (MEDLINE and PubMed) till April 2020 and reference lists of retrieved articles. Only English-language articles were included in the review.

**Results:**

Conservative management with cast immobilization is a safe and successful treatment option in pediatric forearm fractures. Surgical indication is recommended when an acceptable reduction cannot be obtained with closed reduction and casting. Surgical treatment options are intramedullary nail, plating and hybrid fixation.

**Conclusions:**

There is not a unique consensus about fracture management and treatment. Further studies are necessary to create univocal guidelines about optimal treatment, considering new techniques and available technologies.

## Introduction

Forearm fractures are the most common type of fractures in the pediatric population, but, to date, no comprehensive overviews of their epidemiology are available.

Naranje et al. using the 2010 NEISS report, estimated in children aged 0 to 19 years, 5,333,733 emergency room (ER) visits, of which 788,925 (14.7%) were fracture related. Forearm fractures account for 17.8% of all fractures in pediatric age [1].

Joeris et al. [2] found forearm fractures to be significantly more frequent in school age children (65%) and adolescents (63%) compared to infants (42%) and preschool children (50%). Both forearm bones were fractured in 50.1% of cases of forearm injuries and there were significantly more males than females (63.6% vs. 36.4%) [3].

**Fig. 1:**
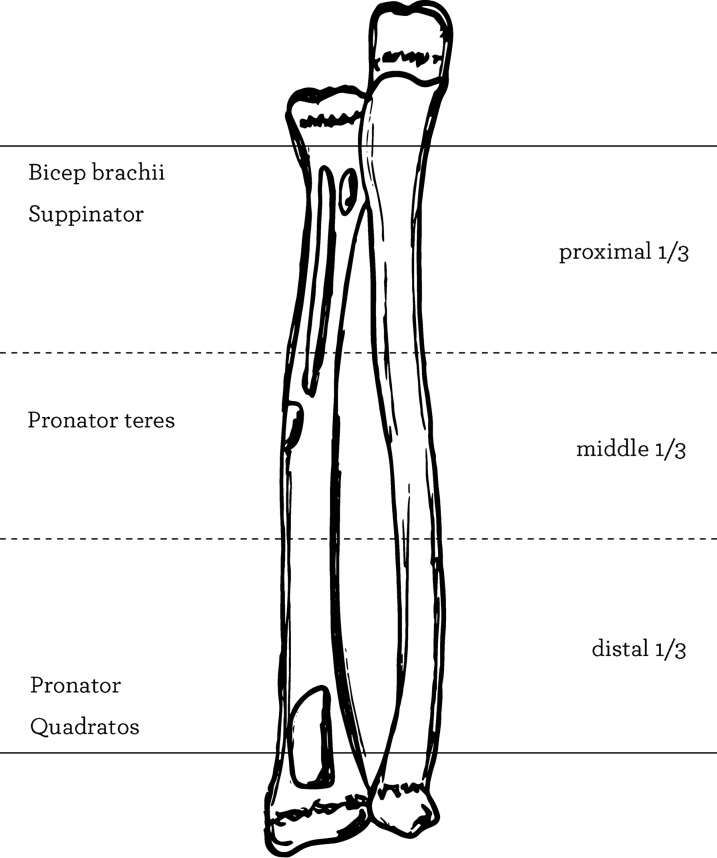
Anatomy of the forearm with the insertion point of the main muscles

Understanding pediatric forearm anatomy offers important guidelines for treatment in the nonoperative and operative settings. Anatomically, the ulna is relatively straight and static, it plays a more important role in maintaining forearm stability, especially when subjected to buckling and torsional stress [4]. Radius and ulna are attached by the proximal annular ligament, by the interosseous membrane along the diaphysis, and distally by the ligaments of the distal radioulnar joint and triangular fibrocartilage complex [5]. The radial bow, an apex lateral bend in the radius, increases the range of pronation [6]. The interosseous membrane is higher strain proximally in neutral and pronation, and is higher strain distally when in supination [7]. The distal radial and ulnar growth plates are responsible for 75% and 81% of the longitudinal growth of each bone, respectively. This polarization of growth shows why distal fractures demonstrate a higher remodeling potential than do fractures closer to the elbow. Additional remodeling can also be attributed to elevation of the thick osteogenic periosteum after fracture [8]. Pediatric forearm fractures typically follow indirect trauma, such as a fall on an outstretched hand coupled with a rotational component [9]. Single bone forearm fractures are far less common and are typically the result of direct trauma. However, single bone forearm fractures of the ulna or radius should always raise suspicion for a Monteggia or Galeazzi fracture dislocation, respectively [10, 11]. Understanding the deforming forces is essential to the reduction in both-bone forearm fractures (Fig. 1). The bicep attaches proximally at the bicipital tuberosity on the anterior medial radius. The supinator and bicep flex and supinate the proximal fragment, when there is a proximal fracture. Fractures that happened in the middle third are altered by the pronator quadratus more distally, which pronates the distal fragment, meantime the impact of bicep on the more proximal fragment is negated by the pronator teres, causing the fragment to remain in neutral position. The brachioradialis dorsiflexes and deviates radially the distal fragment during a distal third fractures [12].

## Diagnosis and classification

Examination of an acute child injured it is not an easy task. Abuse should be considered in children younger than 3-years-old. Distal pulses and capillary refill are assessed. The elbow and wrist are examined to check for a Monteggia or Galeazzi injury. Standard anteroposterior (AP) and lateral orthogonal forearm radiographs are typically sufficient to diagnose a forearm fracture [10]. Although angulation is easier to measure, rotation can be more difficult. New studies evaluate the possibility to use the ultrasound as an accurate method for diagnosis, some advantages could be rapidity, radiation-free and less painful for the patients [13].

On an AP view of an uninjured forearm, the radial styloid will be 180° from the bicipital tuberosity with the tuberosity pointed ulnar. On the lateral view, the ulnar styloid should point posterior, and the coronoid process should point anterior, whereas the radial styloid and the bicipital tuberosity will not be visible [8]. Children bone is softer and more pliable than adults. These properties result in different type of fractures: the buckle (Torus) fracture is characterized by a compression with no disruption of the cortex, the Greenstick fractures characterized by an intact cortical and the other cortex is disrupted on the tension side, the complete fracture is characterized by the involvement of both cortical. A bowing of the radius should be observed for the plasticity of children’s long bones. The Salter and Harris classification is used if the fractures involve the growth plate [14].

## Reduction goals

A correct reduction of a fracture can only be performed by knowing the physiological range of motion of the affected limb. Forearm has a physiological supination of 80° to 120° and physiological pronation of 50° to 80° [15]. Morrey et al. [16] suggest that in the adults only 50° of both pronation and supination is required to task without serious impairment.

In 2019, Valone et al. demonstrate that adolescents utilize more elbow flexion and forearm pronation, with comparatively less supination than children. Awareness of a greater need for specifically flexion and pronation to achieve contemporary tasks such as cell phone and computer use may help guide surgeons in the care of children and adolescents with forearm deformities [17]. Normal function is often achieved with closed reduction and casting. Although reduction does not need to be anatomic because of a child’s ability to remodel, it does need to fall within certain parameters. Most authors [18–20] agree upon the recommendations from Noonan and Price. The acceptable reduction guidelines for pediatric both bone forearm fractures are stratified by age and location and they are summarized in Table 1.

**Table 1:** Acceptable reduction guidelines for pediatric both bone forearm fractures of noonan and price

Patient age	Angulation	Rotation	Bayonet apposition
Age 0–9 (0–8 girls, 0–10 boys)	<15°	<45°	Up to 1 cm
Age >9 (>8 girls, >10 boys)	<10° proximal/midshaft	<30°	Up to 1 cm
Age >9 (>8 girls, >10 boys)	<15° distal	<30°	Up to 1 cm

## Conservative treatment

### Greenstick fractures

Fractures with apex-volar angulation are a result of axial load in supination; therefore, the palm should be rotated volarly (pronation). Fractures with apex-dorsal angulation are a result of pronation force; therefore, the palm should be rotated dorsally (supination).

In case of a greenstick fracture of one bone and a complete fracture of the other one, the same principles of reduction by rotation should be used [21].

After reduction, the forearm should be immobilized in the same position that reduced the fracture. Even if these types of fractures rarely require intervention after initial closed reduction, it is prudent to avoid high-risk activities [22].

Less requirements are needed for buckle fractures that are shown to be more stable than greenstick fractures [23]. For this reason, buckle fractures should be treated with a well-molded below-elbow cast or with the use of a removable wrist immobilizer for 3 weeks, which provides increased early functionality [24].

Some authors suggested that also for minimally angulated greenstick fractures in children <9 years of age splinting is an acceptable alternative [25].

### Complete fractures

**Fig. 2:**
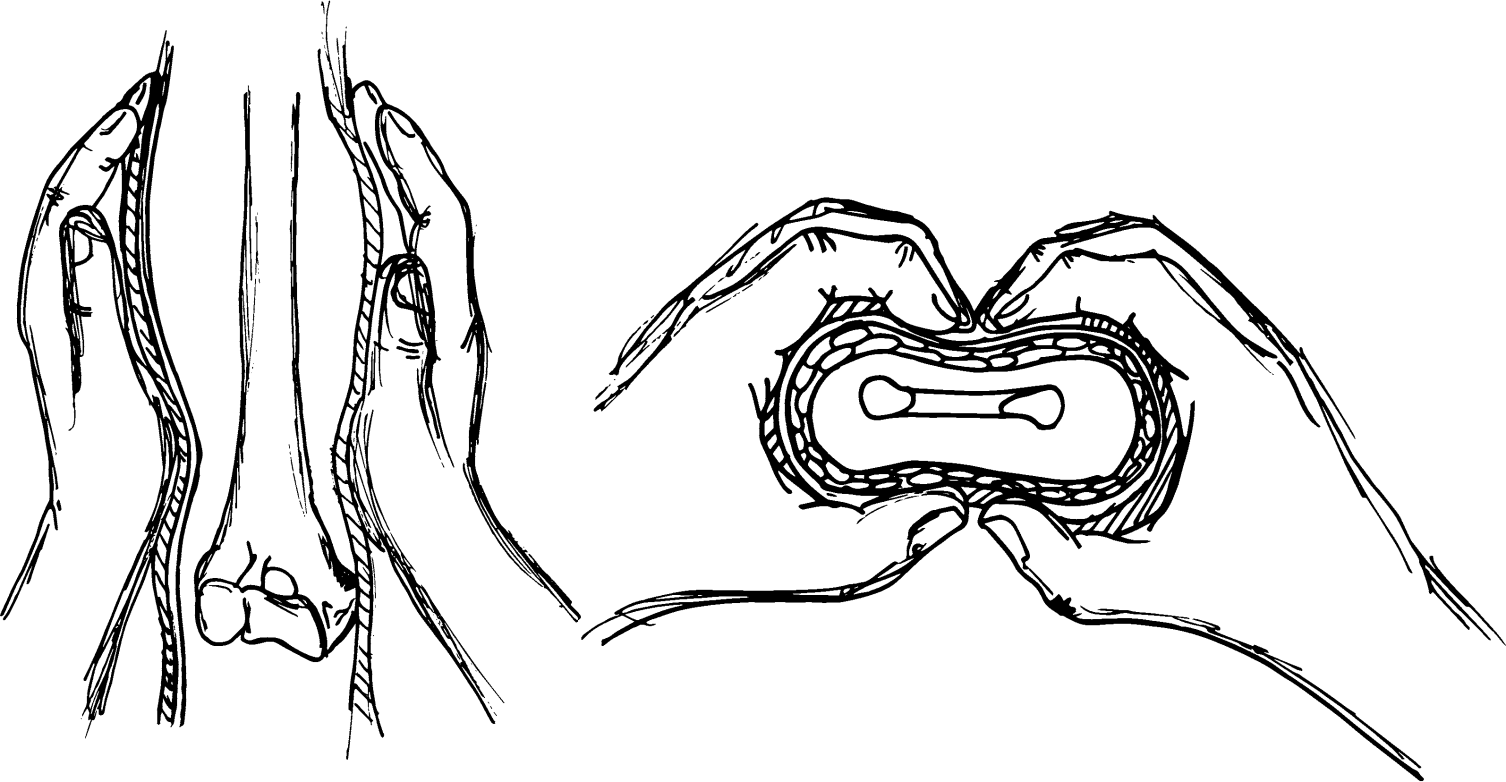
The correct procedure to make a forearm cast is illustrated. Cast is molded with anterior and posterior pressure applied over the intraosseous membrane. Medial and lateral molding above the humeral condyles will prevent distal sliding of the cast

**Fig. 3:**
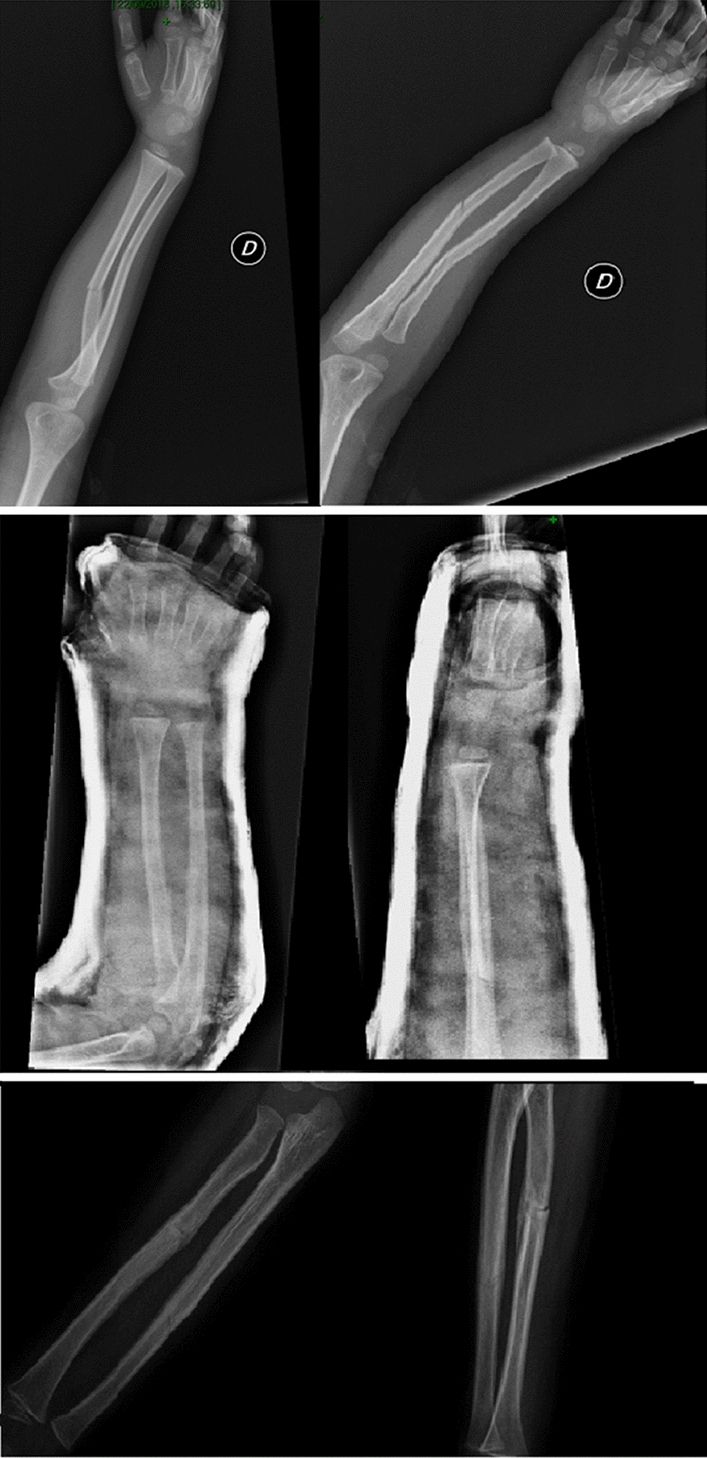
These illustrations show the different stages of conservative management of pediatric both-bone forearm fractures: AP and lateral radiographic projections of the fracture at the time of diagnosis (up), AP and lateral projections after forearm plaster cast (middle), AP and lateral projections after cast removal (down)

Complete both-bone forearm fractures are reduced with a combination of traction and manipulation (Fig. 2). The fingers are plastered to precule skin disease with the elbow at 90° of flexion, and contrary traction is produced [26]. End-to-end apposition is then conducted with direct handling. If alignment is acceptable, the cast is applied and shaped while the arm is still in traction. The hand is positioned in a neutral or modestly supinated posture. All fractures are placed in either fiberglass or plaster long-arm casts with the elbow at 90°. Casts are shaped with anterior and posterior compression claimed over the interosseous membrane. This tends to increase stability in the cast. Medial and lateral plaster above the humeral condyles will preclude the cast from moving distally [8, 27]. Kamat et al*.* demonstrated that a cast index, identified as the ratio of sagittal to coronal diameter of the cast, should be below 0.7 to 0.8. A cast index above this range has been connected with serious risk of lost reduction [20]. A debate is still on set about the distal third of forearm fractures. Webb et al. [28] reported that there were no outcomes differences between short-arm casts and long-arm casts after 8 months of follow-up. After adequate reduction and immobilization, patients typically return for a clinical and radiographic follow-up for the first 3 weeks after injury [18]. It appears that a major chance of failure happens early during nonoperative treatment [19]. Eismann et al. [29] demonstrate that during an average time of 15 days, it is also safe a rereduction after redisplacement following initial closed reduction. Cast immobilization should be maintained for 6–8 weeks, until the clinician finds a complete healing of the fracture. Older children may require 8 to 10 weeks of immobilization. In most cases, patients can resume activities 4–6 weeks after cast removal [5, 30]. Jones et al. examined 300 forearm fractures treated with closed reduction for children from 0 to 8 years of age with angulation over 10 degrees. Only 22 patients require remanipulation. All of these cases resulted on a successful healing of the fractures and did not require any internal fixation [21]. Barvelink et al. [31] show good radiographic and functional outcomes in children with forearm fractures nonreduced and treated with only a mean 28 days of cast, at one year of follow-up. Voto et al. [32] demonstrated that 7% of pediatric forearm fractures treated by cast immobilization had reangulation or displacement. Complications of cast immobilization could be loss of bone mass, muscle atrophy, functional limitations and joint stiffness, that are more severe for adolescent [33, 34]. In conclusion, the analysis of available data in the literature shows that conservative management is a very common, safe and successful treatment option in pediatric forearm fractures (Fig. 3). Stiffness in the elbow and/or wrist is frequent after coming out of the cast. This usually gets better on its own after a few weeks, but in rare cases, physical therapy is needed to help regain motion. Because the bones are still fragile after getting out of the cast, it may recommend that the patient avoids sports and physical education for 4–6 weeks afterwards to prevent the bone from re-breaking. No difference in rehabilitation protocols are proposed in the literature depending on the type of surgical treatment performed [46, 47].

## Operative fixation

Recent articles reported a more surgical management for pediatric and adolescent forearm fractures. Flynn et al. [35] reported a sevenfold increase in surgical management of these fractures. In 2009, Helenius et al. [36] published a review of fractures in pediatric and adolescent age treated at hospitals in Finland between 1997 and 2006. The authors observed an increase of 62% in surgical treatment of forearm fractures occurred during this time compared with previous years. In 2017, Cruz et al. [37] reviewed pediatric forearm fractures treated in the USA between 2000 and 2012. The proportion of fractures treated with surgery improved from 59.3% in 2000 to 70.0% in 2012. Surgery choice was correlated with increasing age, with the lowest rate of surgery occurring in children with 0–4 years old (15.4%) and the highest rate in adolescent with 15–20 years old (79.2%). This trend may be the result of new technology, family and surgeon intolerance of remaining deformity.

## Surgical indication

When an acceptable reduction can not be obtained with closed reduction and casting, operative intervention is recommended. So the greater indications for surgery are unstable and irreducible fractures and furthermore, refracture at a site of previous fracture, open fractures, fractures with neurovascular compromise, pathologic fractures and forearm fractures with associated humerus fracture (“floating elbow”) [38].

Bowman et al. [19] find that those at highest risk are patients 10 years or older, those with proximal-third radius fractures, and ulna fracture angles <15 degrees. These patients should be considered for surgery. Inadequate initial reduction and bayonet apposition with shortening, if the interosseous space is severely compromised, may also be important factors in surgical decision making. It appears that the greatest chance of failure occurs early in nonoperative treatment. So, when this type of fractures occurs at a young age, there is agreement on the type of treatment to be used, and this is the conservative approach according to the indications of Noonan and Price [8]. Instead, for older children there is not a common agreement on the type of treatment to be used, especially in these patients with less than 1 or 2 years of growth remaining, because it worried that the desired degree of remodeling was not achieved before complete skeletal maturity. Surgical intervention is recommended when angulation is >10° in the proximal shaft and midshaft and when 15° in the distal shaft persists after attempts at closed reduction in girls aged >8 years and boys aged >10 years with 2 or more years of remaining growth. Although difficult to quantify, surgery can be considered in the setting of malrotation >30°. Bayonet apposition of any magnitude is not tolerated in older patients [12].

### Intramedullary nails

Intramedullary fixation has become more usual for pediatric forearm fractures needing surgical management [39]. Reasons include small engravings to introduce the fixation device, shorter period of anesthesia and length of hospital stay, safe conservation of the alignment, availability to open and closed fractures and easy removal following placement [12, 40].

There are many studies concluding that flexible intramedullary nailing for the treatment of forearm fractures in children and adolescents is a suitable option [41].

Elastic titanium nails have become the standard of intramedullary (IM) fixation for their biocompatibility, modulus of elasticity, osseointegration rate, corrosion resistance and MRI conformity.

Intramedullary nailing of the forearm is typically acted antegrade for the radius and retrograde for the ulna. Usually, ulna is performed first, as it is accomplished more easily, due to its straight medullary canal [5, 38].

The diameter of available IM implants ranges from 1.5 to 4 mm. The choice depends on the medullary canal. It is usually used a nail that is 40% of the medullary diameter.

**Fig. 4:**
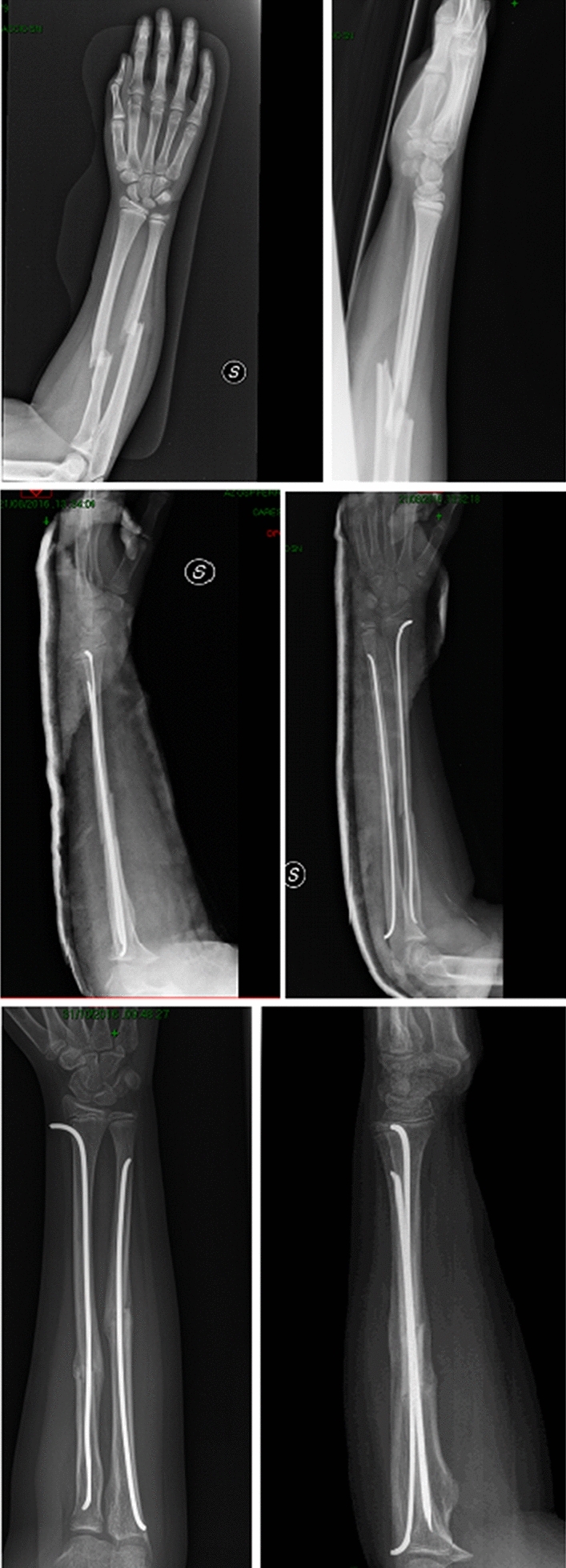
AP and lateral radiographic projection of both-bone forearm fractures treated with double elastic intramedullary nails technique

**Fig. 5:**
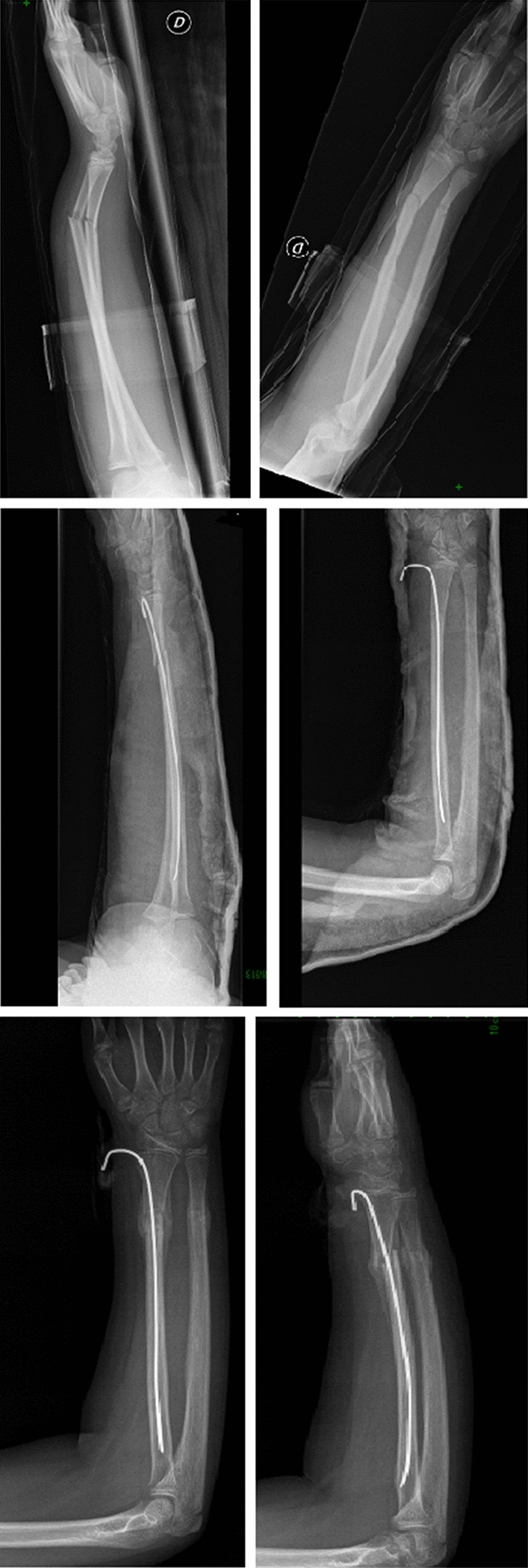
AP and lateral radiographic projection of both-bone forearm fractures treated with single elastic intramedullary nail technique

The use of single versus double nailing technique (Fig. 4) is still a controversial topic. Flynn and Waters [42] were the first to describe the use of single bone intramedullary fixation (Fig. 5) of both bone forearm fractures.

Du et al. [43] scanned 24 children treated with single bone fixation and 25 with double bones fixation and they did not find differences in functional results or complications between the two groups.

Crighton et al. suggest caution in the use of single bone fixation of both bone fracture due to the propensity to increased angulation and progressive deformity of fractures. They state in certain patients, this might be an acceptable treatment option but it is advisable that the fixed bone is anatomically reduced, with particular attention paid to the radial bow. If fixation is required, double bone fixation is generally a construct associated with better outcomes [44].

Yong et al. [45] state that there is not a significant difference in loss of rotation, union time or complications between single bone and both bone fixation.

Herman et al. [5] suggest to use this technique for managing both bone forearm fractures, in which one bone is a greenstick fracture or a easy reducible complete fracture, while the other bone cannot be reduced by casting.

Given the mixed results it would seem that the single-bone fixation is a good option only in younger patients with more distal fractures and higher remodeling capacity.

Duration of postoperative immobilization is variable in the literature ranging from no immobilization and immediate movement to six weeks of long-arm casting [46, 47].

Nails are routinely removed at 6 months, in order bony consolidation to be achieved. Earlier removal is associated with higher refracture rate [48].

The overall complication rate for intramedullary nails ranges from 17 to 42% [10]. They include infection in the site of implant, skin irritation, tendon injury (for example Extensor Pollicis Longus rupture has been reported as a result of friction with sharp nail ends) [38, 49, 50], nerve injury [51], implant migration, bursitis, hypertrophic scars, synostosis, refracture after removal [52], nonunion [38, 53], delayed union [54] and compartment syndrome. This one is associated with longer operative times, longer tourniquet times, open fractures, surgery on the day of injury, younger age [55, 56].

## Plating

**Fig. 6:**
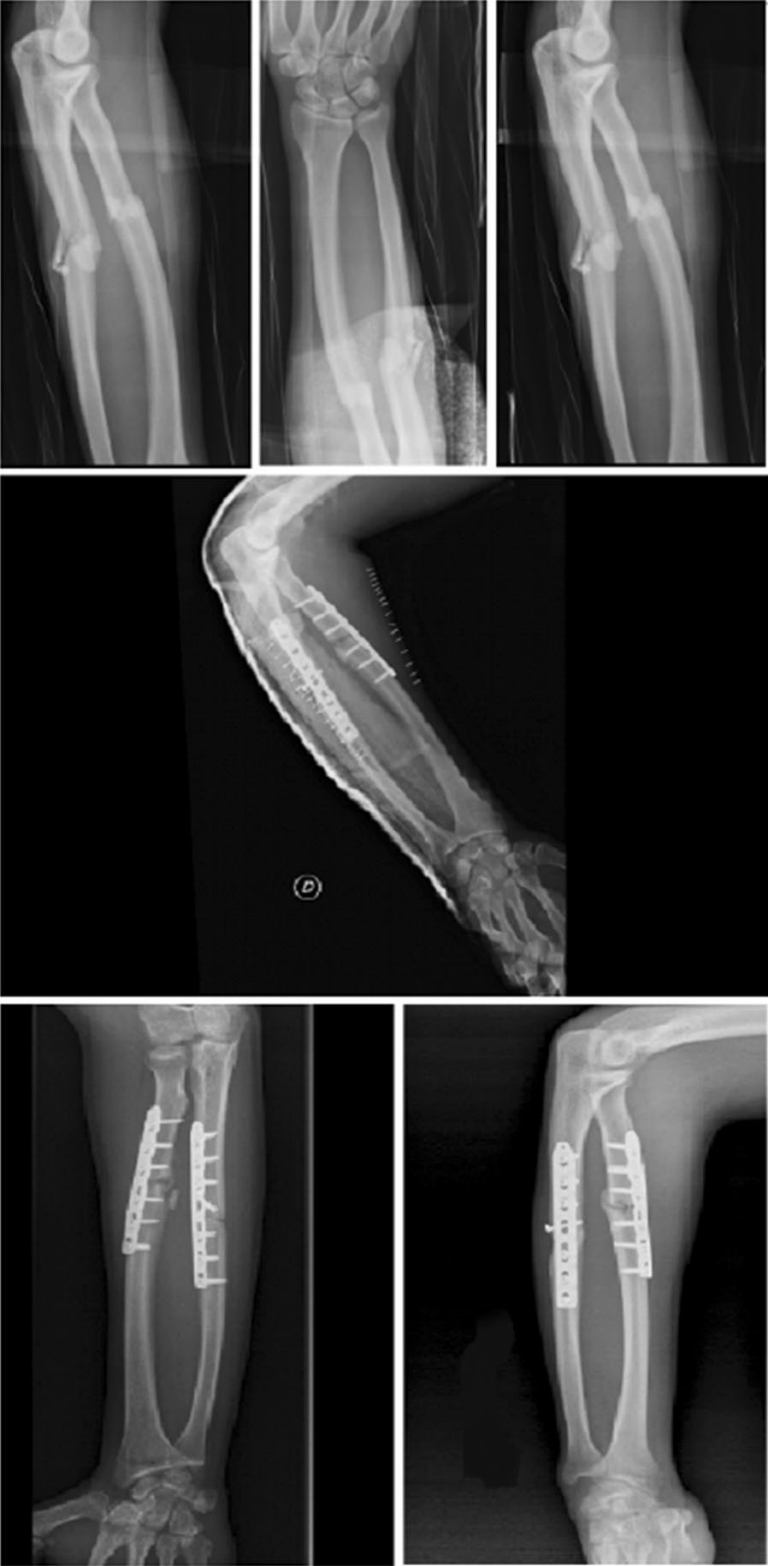
AP and lateral radiographic projections of post-operative treatment of a bi-osseous forearm fractures with two plates

Plating is another valid option for forearm fracture treatment (Fig. 6). Open reduction and use of plate fixation make stabilization and anatomic reduction of forearm fractures, as well as more complete correction of malrotation and restoration radial bow so as to allow early range of motion [57]. However, this approach has been debated as it leads soft-tissue dissection periosteal stripping, needed for exposure and fixation. Plate fixation is indicated in comminuted fractures, fractures on the apex of the radial bow, fractures involving the metaphysis or articular surface, or with late loss of reduction after conservative treatment, in particular in patients who are skeletally mature or with little to no remodeling potential [10, 12, 18, 58].

The right plate choice depends on the patient’s size: a third tubular plate or 2.4-mm compression plate is the most appropriate choice in most children. The length of the plate has to be at least 7 holes to guarantee stability of osteosynthesis, and at least three bicortical screws will be used on both sides of the fracture line [59]. The complication rate for plate fixation range from 16.5 to 33%. Complications include nonunion, malunion, nerve damage, more commonly secondary to ulnar fixation, scar problems, wound infection, hardware failure, refracture [60–62].

The plate removal is debated topic in literature. Vopat et al. [63] showed that leaving the plates in pediatric forearm fractures does not increase the refracture rate compared with the plate removal. Indications for metallic material removal include pain, infection or soft tissue irritation [64].

### Hybrid fixation

Hybrid fixation consists of an elastic stable intramedullary nail for fixing the radius combined with conventional plating for the ulna. It has the goal of helping to preserve radial bow, reduce nonunion rate as well as providing forearm rotational control with ulnar plate fixation while reducing the need for soft tissue dissection for radius plating and refracture rate associated with implant removal.

Zheng et al. [57] stated that hybrid fixation has many advantages over plate screw and intramedullary fixation, including the restoration of the original function of the ulna in the forearm stability. Plate fixation provides better stability than does intramedullary nails fixation, only one important incision, a decreased soft-tissue dissection, placing of a stress-shielding implants on only one fracture and the use of fewer eventually annoying costructions. They concluded that this technique is a sure and efficient therapy for forearm fractures in patients aged 10–16 years. Elhalawany et al. [65] conclude that hybrid fixation technique (plating of ulna and elastic nail for radius) in adolescent forearm fracture seem to be a satisfying option in managing these injuries and seem to reduce the problem of ulnar nonunion encountered when using elastic stable intramedullary nail in that age group. Complications include wound infection, nerve injury, reoperations, non-union. However, studies showed that hybrid fixation has some advantages in terms of the delayed union of the ulna, and the average time of bone union [57].

## What type of fixation should you choose?

**Fig. 7:**
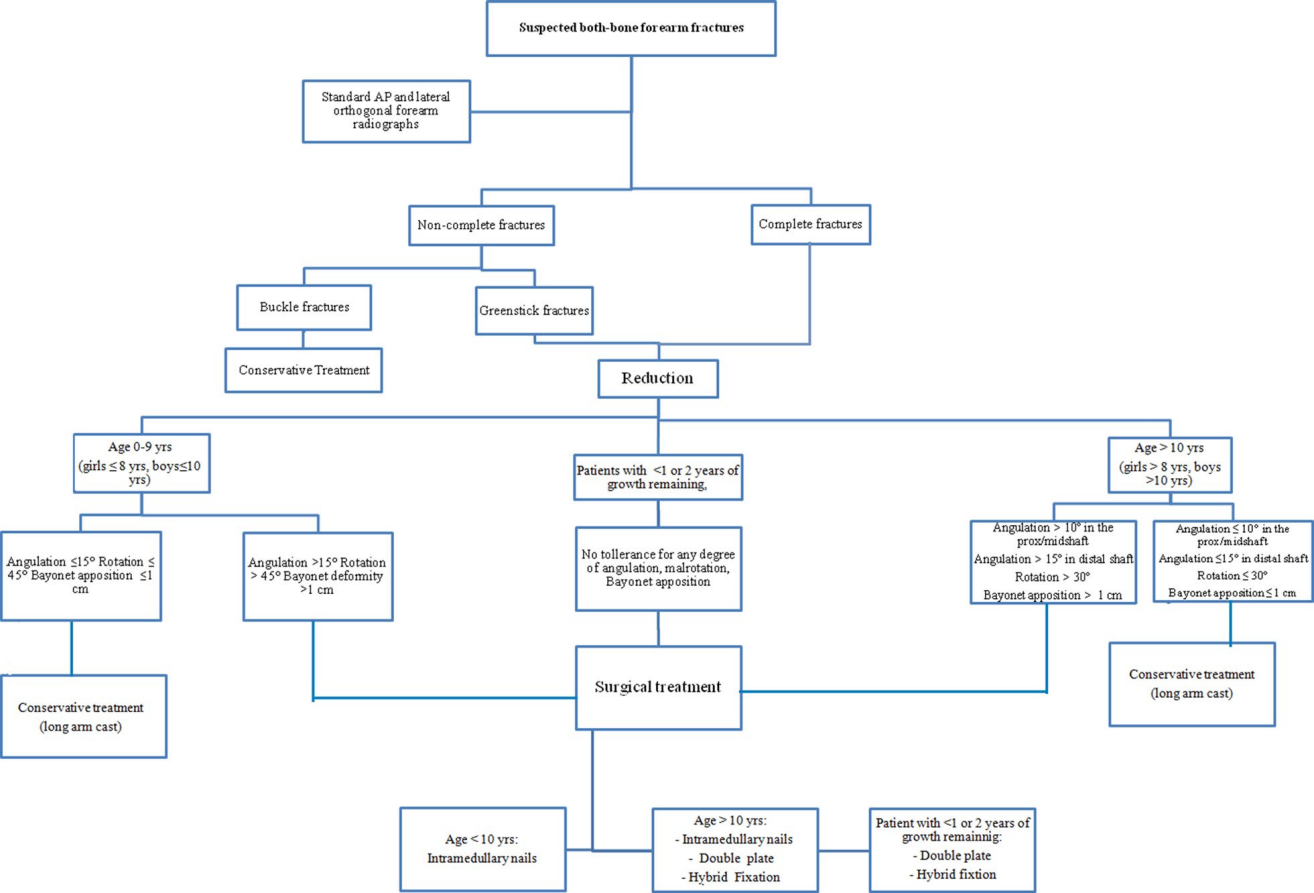
Flowchart treatment proposal, based on the available data in literature

A review of the literature shows similar outcomes of plating and IM nailing in the both forearm fractures in the children. Patel et al. [40] demonstrate no statistical difference in the in functional outcomes and complication rate between plating and IM nailing. Baldwin et al. [62] found similar functional outcomes and complication rate between IM nailing and plate and screw fixation. Freese et al. [60] found 55% of major complications in the IM nails group and no major complications in the plate group. Moreover, they found that the patient treated with IM nailing needed of more time to heal and achieve radiographic union. So they suggest better outcomes and lower complication rate with plate fixation in the adolescent. Westacott et al. [66] suggest that IM nailing may be the elective treatment for simple fracture patterns in order to shorter operative time, better cosmesis and ease of removal. Plating may still have a role in more complex injuries. Based on the available literature we create an operative flowchart in order to help the surgeons for the best treatment decisions (Fig. 7).

## Conclusion

There is not a unique consensus about fracture management and treatment. Current literature agrees about conservative treatment as a gold standard between defined parameters. Exceeded these parameters; the surgical treatment is indicated, with special regard to patients age, fracture pattern and the surgeon experience. Further studies are necessary to create univocal guidelines about optimal treatment, considering new techniques and available technologies.

## References

[1] Naranje SM, Erali RA, Warner WC, Sawyer JR, Kelly DM (2016). Epidemiology of pediatric fractures presenting to emergency departments in the United States. J Pediatr Orthop.

[2] Joeris A, Lutz N, Wicki B, Slongo T, Audigé L (2014). An epidemiological evaluation of pediatric long bone fractures: a retrospective cohort study of 2716 patients from two Swiss tertiary pediatric hospitals. BMC Pediatr.

[3] Ryan LM, Teach SJ, Searcy K, Singer SA, Wood R, Wright JL (2010). Epidemiology of pediatric forearm fractures in Washington. DC J Trauma.

[4] Salvi AE (2006). Forearm diaphyseal fractures: which bone to synthesize first?. Orthopedics.

[5] Herman MJ, Marshall ST (2006). Forearm fractures in children and adolescents: a practical approach. Hand Clin.

[6] Firl M, Wünsch L (2004). Measurement of bowing of the radius. J Bone Joint Surg Br.

[7] Manson TT, Pfaeffle HJ, Herdon JH, Tomaino MM, Fischer KJ (2000). Forearm rotation alters interosseous ligament strain distribution. J Hand Surg.

[8] Noonan KJ, Price CT (1998). Forearm and distal radius fractures in children. J Am Acad Orthop Surg.

[9] Chia B, Kozin SH, Herman MJ, Safier S, Abzug JM (2015). Complications of pediatric distal radius and forearm fractures. Instr Course Lect.

[10] Pace JL (2016). Pediatric and adolescent forearm fractures: current controversies and treatment recommendations. J Am Acad Orthop Surg.

[11] Li Y, James C, Byl N, Sessel J, Caird MS, Farley FA (2020). Obese children have different forearm fracture characteristics compared with normal-weight children. J Pediatr Orthop.

[12] Truntzer J, Vopat ML, Kane PM, Christino MA, Katarincic J, Vopat BG (2015). Forearm diaphyseal fractures in the adolescent population: treatment and management. Eur J Orthop Surg Traumatol Orthop Traumatol.

[13] Epema AC, Spanjer MJB, Ras L, Kelder JC, Sanders M (2019). Point-of-care ultrasound compared with conventional radiographic evaluation in children with suspected distal forearm fractures in the Netherlands: a diagnostic accuracy study. Emerg Med J EMJ.

[14] Randsborg P-H, Sivertsen EA (2009). Distal radius fractures in children: substantial difference in stability between buckle and greenstick fractures. Acta Orthop.

[15] Daruwalla JS (1979). A study of radioulnar movements following fractures of the forearm in children. Clin Orthop.

[16] Morrey BF, Askew LJ, Chao EY (1981). A biomechanical study of normal functional elbow motion. J Bone Joint Surg Am.

[17] Valone LC, Waites C, Tartarilla AB, Whited A, Sugimoto D, Bae DS (2019). Functional elbow range of motion in children and adolescents: J Pediatr Orthop..

[18] Vopat ML, Kane PM, Christino MA, Truntzer J, McClure P, Katarincic J (2014). Treatment of diaphyseal forearm fractures in children. Orthop Rev.

[19] Bowman EN, Mehlman CT, Lindsell CJ, Tamai J (2011). Non-operative treatment of both-bone forearm shaft fractures in children: predictors of early radiographic failure. J Pediatr Orthop.

[20] Kamat AS, Pierse N, Devane P, Mutimer J, Horne G (2012). Redefining the cast index: the optimum technique to reduce redisplacement in pediatric distal forearm fractures. J Pediatr Orthop.

[21] Jones K, Weiner DS (1999). The management of forearm fractures in children: a plea for conservatism. J Pediatr Orthop.

[22] Ting BL, Kalish LA, Waters PM, Bae DS (2016). Reducing cost and radiation exposure during the treatment of pediatric greenstick fractures of the forearm. J Pediatr Orthop.

[23] Symons S, Rowsell M, Bhowal B, Dias JJ (2001). Hospital versus home management of children with buckle fractures of the distal radius. A prospective, randomised trial. J Bone Joint Surg Br.

[24] Primavesi R (2011). Sticks and stones and broken bones. Can Fam Physician.

[25] Runyon RS, Doyle SM (2017). When is it ok to use a splint versus cast and what remodeling can one expect for common pediatric forearm fractures. Curr Opin Pediatr.

[26] Carey PJ, Alburger PD, Betz RR, Clancy M, Steel HH (1992). Both-bone forearm fractures in children. Orthopedics.

[27] Pretell Mazzini J, Rodriguez Martin J (2010). Paediatric forearm and distal radius fractures: risk factors and re-displacement-role of casting indices. Int Orthop.

[28] Webb GR, Galpin RD, Armstrong DG (2006). Comparison of short and long arm plaster casts for displaced fractures in the distal third of the forearm in children. J Bone Joint Surg Am.

[29] Eismann EA, Parikh SN, Jain VV (2016). Rereduction for redisplacement of both-bone forearm shaft fractures in children. J Pediatr Orthop.

[30] Wilson JM, London NJ, Limb D (2000). A new fracture of the forearm adjacent to a healing fracture. Int Orthop.

[31] Barvelink B, Ploegmakers JJW, Harsevoort AGJ, Stevens M, Verheyen CC, Hepping AM (2020). The evolution of hand function during remodelling in nonreduced angulated paediatric forearm fractures: a prospective cohort study. J Pediatr Orthop B.

[32] Voto SJ, Weiner DS, Leighley B (1990). Redisplacement after closed reduction of forearm fractures in children. J Pediatr Orthop.

[33] Ceroni D, Martin X, Delhumeau-Cartier C, Rizzoli R, Kaelin A, Farpour-Lambert N (2012). Is bone mineral mass truly decreased in teenagers with a first episode of forearm fracture? A prospective longitudinal study. J Pediatr Orthop.

[34] Boero S, Michelis MB, Calevo MG, Stella M (2007). Multiple forearm diaphyseal fracture: reduction and plaster cast control at the end of growth. Int Orthop.

[35] Flynn JM, Jones KJ, Garner MR, Goebel J (2010). Eleven years experience in the operative management of pediatric forearm fractures. J Pediatr Orthop.

[36] Helenius I, Lamberg TS, Kääriäinen S, Impinen A, Pakarinen MP (2009). Operative treatment of fractures in children is increasing. A population-based study from Finland. J Bone Joint Surg Am.

[37] Cruz AI, Kleiner JE, DeFroda SF, Gil JA, Daniels AH, Eberson CP (2017). Increasing rates of surgical treatment for paediatric diaphyseal forearm fractures: a national database study from 2000 to 2012. J Child Orthop.

[38] Poutoglidou F, Metaxiotis D, Kazas C, Alvanos D, Mpeletsiotis A (2020). Flexible intramedullary nailing in the treatment of forearm fractures in children and adolescents, a systematic review. J Orthop.

[39] Cumming D, Mfula N, Jones JWM (2008). Paediatric forearm fractures: the increasing use of elastic stable intra-medullary nails. Int Orthop.

[40] Patel A, Li L, Anand A (2014). Systematic review: functional outcomes and complications of intramedullary nailing versus plate fixation for both-bone diaphyseal forearm fractures in children. Injury.

[41] Wall L, O’Donnell J, Schoenecker P, Keeler K, Dobbs M, Luhmann S (2012). Titanium elastic nailing radius and ulna fractures in adolescents. J Pediatr Orthop B.

[42] Flynn JM, Waters PM (1996). Single-bone fixation of both-bone forearm fractures. J Pediatr Orthop.

[43] Du S-H, Feng Y-Z, Huang Y-X, Guo X-S, Xia D-D (2016). Comparison of pediatric forearm fracture fixation between single- and double-elastic stable intramedullary nailing. Am J Ther.

[44] Crighton EA, Huntley JS (2018). Single versus double intramedullary fixation of paediatric both bone forearm fractures: radiological outcomes. Cureus.

[45] Yong B, Yuan Z, Li J, Li Y, Southern EP, Canavese F (2018). Single bone fixation versus both bone fixation for pediatric unstable forearm fractures: a systematic review and metaanalysis. Indian J Orthop.

[46] Lascombes P, Haumont T, Journeau P (2006). Use and abuse of flexible intramedullary nailing in children and adolescents. J Pediatr Orthop.

[47] Shah AS, Lesniak BP, Wolter TD, Caird MS, Farley FA, Vander Have KL (2010). Stabilization of adolescent both-bone forearm fractures: a comparison of intramedullary nailing versus open reduction and internal fixation. J Orthop Trauma.

[48] Lyman A, Wenger D, Landin L (2016). Pediatric diaphyseal forearm fractures: epidemiology and treatment in an urban population during a 10-year period, with special attention to titanium elastic nailing and its complications. J Pediatr Orthop B.

[49] Lee AK, Beck JD, Mirenda WM, Klena JC (2016). Incidence and risk factors for extensor pollicis longus rupture in elastic stable intramedullary nailing of pediatric forearm shaft fractures. J Pediatr Orthop.

[50] Murphy HA, Jain VV, Parikh SN, Wall EJ, Cornwall R, Mehlman CT (2019). Extensor tendon injury associated with dorsal entry flexible nailing of radial shaft fractures in children: a report of 5 new cases and review of the literature. J Pediatr Orthop.

[51] Nørgaard SL, Riber SS, Danielsson FB, Pedersen NW, Viberg B (2018). Surgical approach for elastic stable intramedullary nail in pediatric radius shaft fracture: a systematic review. J Pediatr Orthop Part B.

[52] Han B, Wang Z, Li Y, Xu Y, Cai H (2019). Risk factors for refracture of the forearm in children treated with elastic stable intramedullary nailing. Int Orthop.

[53] Ogonda L, Wong-Chung J, Wray R, Canavan B (2004). Delayed union and non-union of the ulna following intramedullary nailing in children. J Pediatr Orthop B.

[54] Lobo-Escolar A, Roche A, Bregante J, Gil-Alvaroba J, Sola A, Herrera A (2012). Delayed union in pediatric forearm fractures. J Pediatr Orthop.

[55] Blackman AJ, Wall LB, Keeler KA, Schoenecker PL, Luhmann SJ, O’Donnell JC (2014). Acute compartment syndrome after intramedullary nailing of isolated radius and ulna fractures in children. J Pediatr Orthop.

[56] Martus JE, Preston RK, Schoenecker JG, Lovejoy SA, Green NE, Mencio GA (2013). Complications and outcomes of diaphyseal forearm fracture intramedullary nailing: a comparison of pediatric and adolescent age groups. J Pediatr Orthop.

[57] Zheng W, Tao Z, Chen C, Zhang C, Zhang H, Feng Z (2018). Comparison of three surgical fixation methods for dual-bone forearm fractures in older children: a retrospective cohort study. Int J Surg Lond Engl.

[58] Pretell-Mazzini J, Zafra-Jimenez JA, Rodriguez Martin J (2010). Clinical application of locked plating system in children. An orthopaedic view. Int Orthop.

[59] Chen C, Xie L, Zheng W, Chen H, Cai L (2019). Evaluating the safety and feasibility of a new surgical treatment for forearm fractures in older children: study protocol for a randomised controlled trial. Trials.

[60] Freese KP, Faulk LW, Palmer C, Baschal RM, Sibbel SE (2018). A comparison of fixation methods in adolescent patients with diaphyseal forearm fractures. Injury.

[61] Lee SK, Kim KJ, Lee JW, Choy WS (2014). Plate osteosynthesis versus intramedullary nailing for both forearm bones fractures. Eur J Orthop Surg Traumatol Orthop Traumatol.

[62] Baldwin K, Morrison MJ, Tomlinson LA, Ramirez R, Flynn JM (2014). Both bone forearm fractures in children and adolescents, which fixation strategy is superior: plates or nails? A systematic review and meta-analysis of observational studies. J Orthop Trauma.

[63] Vopat BG, Kane PM, Fitzgibbons PG, Got CJ, Katarincic JA (2014). Complications associated with retained implants after plate fixation of the pediatric forearm. J Orthop Trauma.

[64] Boulos A, DeFroda SF, Kleiner JE, Thomas N, Gil JA, Cruz AI (2017). Inpatient orthopaedic hardware removal in children: a cross-sectional study. J Clin Orthop Trauma.

[65] Elhalawany AS, Afifi A, Anbar A, Galal S (2020). Hybrid fixation for adolescent both-bones diaphyseal forearm fractures: preliminary results of a prospective cohort study. J Clin Orthop Trauma.

[66] Westacott DJ, Jordan RW, Cooke SJ (2012). Functional outcome following intramedullary nailing or plate and screw fixation of paediatric diaphyseal forearm fractures: a systematic review. J Child Orthop.

